# A cost-effectiveness and budget impact analysis of first-line fidaxomicin for patients with *Clostridium difficile* infection (CDI) in Germany

**DOI:** 10.1007/s15010-016-0894-y

**Published:** 2016-04-09

**Authors:** Maureen Watt, Charles McCrea, Sukhvinder Johal, John Posnett, Jameel Nazir

**Affiliations:** 1 Astellas EMEA, Chertsey, UK; 2 PAREXEL Access Consulting, London, UK; 3 Astellas Pharma Europe Ltd., 2000 Hillswood Drive, Chertsey, KT16 0RS UK

**Keywords:** *Clostridium difficile*, Cost-effectiveness, Budget impact, Fidaxomicin, Vancomycin

## Abstract

**Purpose:**

*Clostridium difficile* infection (CDI) represents a significant economic healthcare burden, especially the cost of recurrent disease. Fidaxomicin produced significantly lower recurrence rates and higher sustained cure rates in clinical trials. We evaluated the cost-effectiveness and budget impact of fidaxomicin compared with vancomycin in Germany in the first-line treatment of patient subgroups with CDI at increased risk of recurrence.

**Methods:**

A semi-Markov model was used to compare the cost-effectiveness and budget impact of fidaxomicin vs. vancomycin from a payer perspective in Germany. The model cycle length was 10 days. The time horizon was 1 year. Model inputs were probability of clinical cure, 30-day probability of recurrence, and 30-day attributable mortality based on evidence from two randomized controlled trials comparing fidaxomicin and vancomycin in patients with CDI. Cost-effectiveness outcomes were cost per quality-adjusted life year gained, cost per bed-day saved, and cost per recurrence avoided.

**Results:**

Despite higher drug acquisition costs, fidaxomicin was dominant in the cancer subgroup (less costly and more effective) and cost-effective in the other subgroups, with incremental cost-effectiveness ratios vs. vancomycin ranging from €26,900 to €44,500. Hospitalization costs of the first-line treatment of CDI with fidaxomicin vs. vancomycin were lower in every patient subgroup, resulting in budget impacts ranging from −€1325 (in patients ≥65 years) to −€2438 (in cancer patients). Reductions in the cost of treating recurrence with fidaxomicin ranged from −€574.32 per patient in those receiving concomitant antibiotics to −€1500.68 per patient in renally impaired patients.

**Conclusions:**

In patient subgroups with CDI at increased recurrence risk, fidaxomicin was cost-effective vs. vancomycin, and less costly and more effective in patients with cancer.

**Electronic supplementary material:**

The online version of this article (doi:10.1007/s15010-016-0894-y) contains supplementary material, which is available to authorized users.

## Introduction

*Clostridium difficile* infection (CDI) is a debilitating condition associated with mortality, substantial morbidity, and hospitalization [1, 2]. Treatment options for CDI patients have been vancomycin and metronidazole for a number of years. However, CDI recurs in approximately 20–25 % of patients treated with these agents [3, 4]. Recurrent CDI places a heavy burden on patients, including prolonged symptoms, repeated courses of antibiotics, and the attendant risk of side-effects and rehospitalization [5]. Certain subgroups of patients are more susceptible to recurrence, e.g., those with severe CDI, cancer or renal impairment, those with a previous recurrence, those aged ≥65 years, and those receiving concomitant antibiotics [6–9]. Severe CDI and patients with recurrence are recognized as identifiable patient subgroups in the real-world clinical setting by ESCMID guidelines [10]; elderly patients are at higher risk of severe or severe-complicated CDI [11]. Patients with cancer, with impaired renal function or receiving concomitant antibiotics have been associated with lower cure rates and longer time to resolution of diarrhea [7, 12, 13].

CDI also represents a significant economic healthcare burden due to the costs associated with increased length of hospital stay [14]. Moreover, the costs associated with recurrent CDI may be greater than those associated with the initial episode, not only as a result of longer hospital stay, but also the need for environmental decontamination, rigorous hygiene in patient care, and in some cases, cohort isolation and ward closure [5]. Indeed, a recent US study confirmed a greater healthcare utilization and mortality in patients with recurrent CDI compared with non-recurrent disease [15]. Moreover, the direct treatment costs of *C. difficile*-associated diarrhea (CDAD) in a German hospital were recently estimated at €73,898 per patient with ≥1 recurrence [16]. Clearly, the ability to decrease the risk of recurrent CDI is likely to benefit patients, by reducing morbidity and mortality, and also healthcare systems, by reducing costs.

Fidaxomicin is the first in a new class of macrocyclic antibiotics licensed to treat CDI. In two phase III trials, fidaxomicin was shown to be non-inferior to vancomycin in terms of clinical cure and produced significantly lower recurrence rates and significantly higher sustained cure rates [3, 17]. In patients with severe CDI and those with a first CDI recurrence, recent studies in Scotland and Ireland, respectively, have shown that fidaxomicin was cost-effective compared with vancomycin [18] and less costly and more effective than vancomycin or metronidazole [19].

In Europe, the annual management costs of CDI were estimated at around €3 billion, and since CDAD occurs predominantly among the elderly, this is expected to increase in future as the proportion of elderly people in the European population increases [20]. In Germany, CDAD has been associated with an annual cost burden of €464 million for the German healthcare system [21]. This study evaluates the cost-effectiveness and budget impact of fidaxomicin compared with vancomycin in Germany in the first-line treatment of patient subgroups with CDI at increased risk of recurrence.

## Methods

### Model overview

**Fig. 1:**
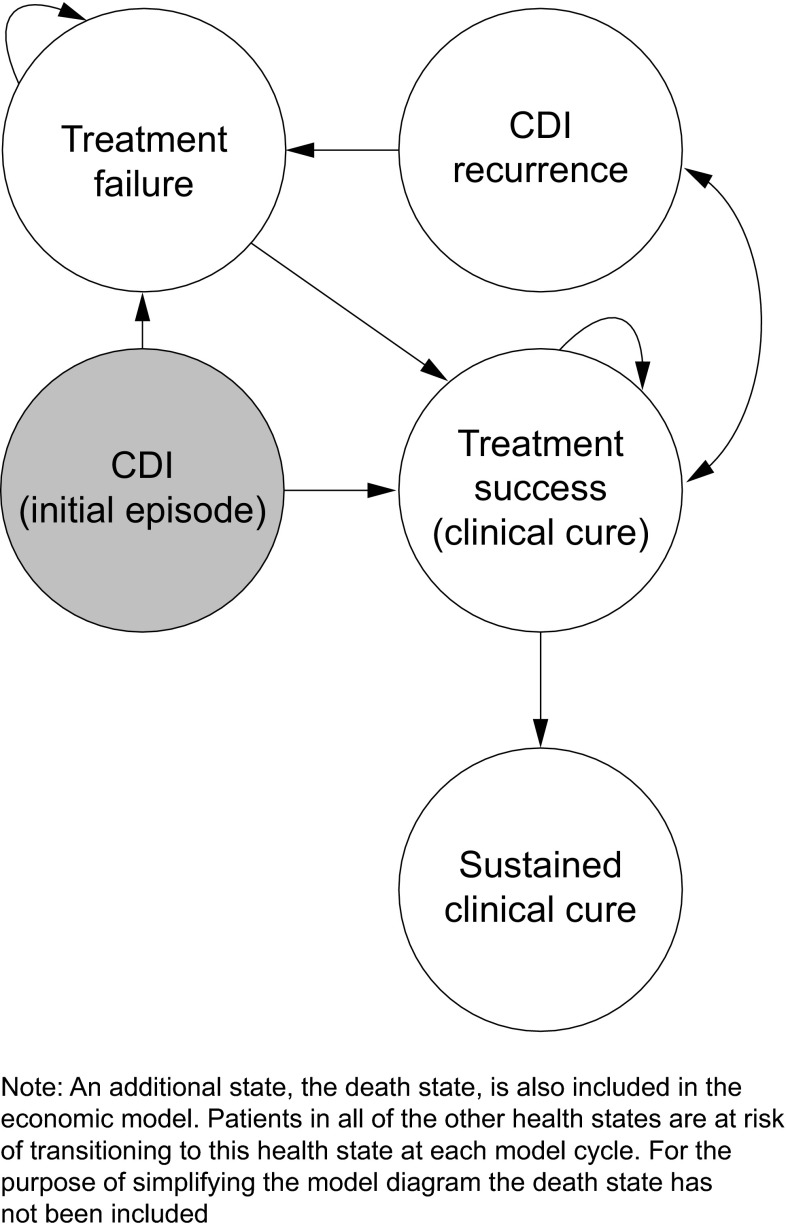
Markov model structure used to assess the cost-effectiveness of first-line fidaxomicin vs. vancomycin in patients with CDI in Germany. *CDI* *Clostridium difficile *infection

A semi-Markov model was developed in Microsoft Excel to simulate the disease course and therapeutic management in patients with CDI. The model evaluated the cost-effectiveness and budget impact of fidaxomicin vs. vancomycin as the first-line treatment of patient subgroups with CDI at increased recurrence risk from a payer perspective in Germany. The model cycle length was 10 days (corresponding to the duration of a course of treatment in clinical practice). The time horizon was 1 year to account for multiple recurrences. The base-case model was populated with cost data for Germany. No discounting was applied to costs or outcomes, because the time horizon was 1 year. The model framework was based on information gathered from existing clinical guidelines and the available published clinical efficacy data for each treatment. The structure of the model is shown in Fig. 1. It was applied to the following patient subgroups, who are more susceptible to recurrence [6–9]: patients with ≥1 recurrence; severe CDI; those receiving concomitant antibiotics; age ≥65 years; patients with cancer; and patients with renal failure.

The cost-effectiveness analysis was evaluated for a cohort of 100 patients. Cost-effectiveness outcomes were expressed as the cost per quality-adjusted life year (QALY) gained, cost per bed-day saved, and the cost per recurrence avoided. Irrespective of subgroup, patients entered the model in the ‘Initial CDI episode’ state and were treated with either fidaxomicin 200 mg twice daily or vancomycin 125 mg four times daily for 10 days (Treatment 1). Successfully treated patients entered and remained in the ‘CDI cured’ health state (Fig. 1). If a patient had a recurrence, they then moved back to the ‘Initial CDI episode’ state and were treated again with the initial treatment. If patients did not achieve clinical cure with initial treatment, they received a second 10-day course of treatment with vancomycin or fidaxomicin (Treatment 2). If patients were still not cured after the second-line therapy, they received third-line ‘rescue’ treatment. Patients who were not cured had a risk of CDI-attributable mortality.

### Assumptions

Irrespective of initial treatment, in the base-case, the second-line treatment (Treatment 2) for treatment failures was vancomycin. ‘Rescue’ treatment (fecal implant) was assumed to have a 100 % cure rate, with an assumed cost of £1500. This is a representative cost only and does not imply that all patients will have this third-line rescue treatment. The third-line rescue treatment is used after two treatment failures; only a small percentage of patients overall (1.6–2.4 %) go on to receive rescue treatment (Table 12 Supplementary Data). It was assumed that all cases (initial episode, treatment failures, and recurrences) were treated in hospital. In addition, the risk of recurrence (all patients) and CDI-attributable mortality (non-cured patients only) was applied for 30 days following each initial treatment cycle.

### Inputs

#### Efficacy

Model inputs were probability of clinical cure, 30-day probability of recurrence after the end of treatment, and 30-day CDI-attributable mortality (Table 1). Clinical cure was defined as the resolution of diarrhea with maintenance of resolution and no further requirement for CDI therapy on the second day after the end of therapy (10 days +2). Recurrence was defined as: reappearance of ≥3 diarrheal stools per 24-h period within 4 weeks (28 days) after cessation of therapy; the presence of *C. difficile* toxin A, B, or both in the stools; and the need for retreatment for CDI following resolution of diarrhea. Model inputs were based on data from two phase III clinical trials comparing fidaxomicin and vancomycin in patients with CDI [3, 9, 12, 17]. Recurrence rates for patients with cancer and patients with renal impairment were derived from Cornely et al. [12] and Mullane et al. [13].

**Table 1:** Clinical efficacy inputs for the model (pooled data from two phase III clinical trials comparing fidaxomicin and vancomycin in patients with CDI [3, 9, 12, 17])

	Fidaxomicin	Vancomycin
**Clinical cure rate (%)**		
≥1 recurrence	89.8	88.9
Severe CDI	80.0	82.6
Concomitant antibiotics	84.3	75.5
≥65 years	84.8	84.5
Cancer	85.1	74.0
Renal impairment	73.9	76.0
**Recurrence rate (%)**		
≥1 recurrence	20.3	32.5
Severe CDI	11.4	28.3
Concomitant antibiotics	17.4	25.5
≥65 years	16.1	29.3
Cancer	13.5	29.6
Renal impairment	14.7	31.6
**Mortality rate (%)**		
All subgroups	6.5	6.5

*CDI* *Clostridium difficile* infection

According to the model, clinical cure rates were generally similar between treatments, although rates were slightly higher with fidaxomicin in patients receiving concomitant antibiotics and in those with cancer (Table 1). Recurrence rates were lower with fidaxomicin (11.4–20.3 %) than vancomycin (25.5–32.5 %) in all patient subgroups. In the model, the same recurrence risk was applied to first and subsequent recurrences.

The same mortality rate (6.5 %) was applied to all patient subgroups. This was based on 30-day all-cause mortality from the comparative phase III trials (6.5 %) [3, 17] and is based on the assumption that 30-day all-cause mortality is a reasonable proxy for CDI-attributable mortality. Indeed, the rate used is consistent with the literature estimates of CDI-attributable mortality (5.7–6.9 %) [22–24].

#### Safety

No treatment-related adverse events were considered, because these were assumed to be mild, based on data from clinical trials, and it was assumed that they would not lead to additional treatment costs or a switch in treatment.

#### Costs

All costs were expressed in €, for the year 2014. Only direct medical costs were considered (the costs of treatment with fidaxomicin or vancomycin, and costs of hospitalization). The following drug acquisition costs (correct at Q1 2014) were used [25]:

- 10-day course of fidaxomicin: €1387.
- 10-day course of vancomycin: €61.
- Rescue treatment (fecal implant): €1500 [26].

Hospitalization costs were based on a cost per day of €348 on a general ward and an average length of stay of 14.6 days (derived from the German drug tariff [Institute for the Hospital Remuneration System 2014] for DRG codes in which a high proportion of cases was listed under code A04.7, ‘Enterocolitis due to *Clostridium difficile*’); these include the costs of both materials and personnel. In the base-case setting, length of stay was the same for a recurrence as for the initial episode.

#### Utilities

The following health state utility values [27] were used in the model: 0.33 per cycle for patients with CDI; 0.56 for patients treated successfully (first cycle, 10 days) after the end of treatment; 0.78 for successfully treated patients (second and subsequent cycles after the end of treatment); and 0.78 for patients without CDI. Due to a lack of available CDI-specific utility data, the data utility source used was not disease-specific; however, these utility data have been used in other published cost-effectiveness and cost-utility CDI models [18, 19].

#### Sensitivity analyses

One-way deterministic sensitivity analyses were carried out to test the robustness of the model outcomes and the effect of changing each variable in the model separately. Each variable used in the base-case was varied by ±20 %, except for the ‘probability of clinical cure’, which is varied by ±10 % to avoid the probability of being greater than 100 %. The use of the univariate and best- or worst-case sensitivity analyses is an important way of identifying parameters that may have a substantial impact on the cost-effectiveness results and of explaining the key drivers of the model [28]. The cost of rescue treatment and percentage of patients receiving rescue treatment were tested in sensitivity analyses; since such a small percentage of patients receive this therapy, this parameter had very little effect on cost-effectiveness outcomes.

## Results

### Cost-effectiveness analyses

Despite higher drug acquisition costs, fidaxomicin was dominant in the cancer subgroup (less costly and more effective) and cost-effective in the other subgroups, with incremental cost-effectiveness ratios (ICERs; incremental cost per QALY gained) vs. vancomycin ranging from €26,900 to €44,500 (based on a national willingness-to-pay threshold of €50,000) [29] (Table 2). Fidaxomicin was associated with incremental costs per bed-day saved ranging from €71 to €121 and costs per recurrence avoided ranging from €1247 to €2600 (or dominant in the cancer subgroup on both measures).

**Table 2:** Cost-effectiveness results for the first-line treatment of CDI in 100 patients treated with fidaxomicin vs. vancomycin in patient subgroups

Group	Incremental cost (€)	Incremental QALYs	Cost per bed-day saved (€)	Cost per recurrence avoided (€)	ICER (vs. vancomycin)	CEA outcome
≥1 recurrence	46,079	1.05	119	2049	€43,900/QALY gained	Cost-effective
Severe CDI	39,613	1.14	99	1532	€34,800/QALY gained	Cost-effective
Concomitant antibiotics	29,080	0.95	71	2600	€30,700/QALY gained	Cost-effective
≥65 years	46,116	1.04	121	2127	€44,500/QALY gained	Cost-effective
Cancer	−80,621	1.64	Fidaxomicin dominant	Fidaxomicin dominant	Fidaxomicin dominant	Fidaxomicin dominant
Renal impairment	33,403	1.24	73	1,247	€26,900/QALY gained	Cost-effective

*CDI Clostridium difficile* infection, *CEA* cost-effectiveness analysis, *ICER* incremental cost-effectiveness ratio, *QALY* quality-adjusted life year, *ICER* values are rounded to the nearest 100

### Budget impact analyses

The cost per patient derived from the cost-effectiveness analysis was used to estimate the budget impact of replacing vancomycin with fidaxomicin in the first-line treatment of CDI. The 1-year net budget impact per patient of using first-line fidaxomicin rather than vancomycin in each of the patient subgroups is shown in Table 3. For each subgroup, the total cost associated with first-line fidaxomicin was higher than vancomycin, with the exception of patients with cancer, where the budget impact of fidaxomicin was €806 lower per patient. The total costs consist of the cost of medication and the cost of hospitalization (Table 4). Hospitalization costs of the first-line treatment of CDI with fidaxomicin vs. vancomycin were lower in every patient subgroup, resulting in budget impacts ranging from −€1325 (in patients ≥65 years) to −€2438 (in cancer patients).

**Table 3:** Budget impact (per patient) of the first-line treatment of CDI with fidaxomicin vs. vancomycin in patient subgroups

Subgroups	Total costs associated with first-line fidaxomicin (€)	Total costs associated with first-line vancomycin (€)	Budget impact (€)
≥1 recurrence	9016	8555	461
Severe CDI	8987	8591	396
Concomitant antibiotics	9202	8911	291
≥65 years	9008	8547	461
Cancer	8726	9532	−806
Renal impairment	9927	9593	334

*CDI* *Clostridium difficile* infection

**Table 4:** Hospitalization costs (per patient) of the first-line treatment of CDI with fidaxomicin vs. vancomycin in patient subgroups

Subgroups	Hospitalization costs associated with first-line fidaxomicin (€)	Hospitalization costs associated with first-line vancomycin (€)	Budget impact (€)
≥1 recurrence	7081	8429	−1347
Severe CDI	7056	8432	−1376
Concomitant antibiotics	7226	8698	−1472
≥65 years	7074	8399	−1325
Cancer	6853	9291	−2438
Renal impairment	7792	9367	−1575

*CDI* *Clostridium difficile* infection

Table 5 shows that when patients are treated with fidaxomicin, although the cost of medication is higher, there are considerable savings due to the reduction in recurrences, compared with vancomycin in all patient subgroups. Reductions in the cost of treating recurrence with fidaxomicin ranged from −€574.32 per patient in those receiving concomitant antibiotics to −€1500.68 per patient in renally impaired patients. Reduction in the number of recurrences with fidaxomicin ranged from −0.1118 to −0.2678 per patient, respectively.

**Table 5:** Economic and patient impact of less recurrence with fidaxomicin, compared with vancomycin in 100 patients treated with fidaxomicin vs. vancomycin

Subgroups	Reduction in the cost of treating recurrence with fidaxomicin vs. vancomycin (€)	Reduction in the number of recurrences with fidaxomicin vs. vancomycin
≥1 recurrence	−94,129	−22.49
Severe CDI	−138,150	−25.86
Concomitant antibiotics	−57,432	−11.18
≥65 years	−103,349	−21.68
Cancer	−148,142	−23.06
Renal impairment	−150,068	−26.78

*CDI* *Clostridium difficile* infection

### Sensitivity analyses

The deterministic sensitivity analyses showed that the key drivers of cost-effectiveness were the rate of recurrence, clinical cure rate, and CDI-attributable mortality rate. Details of the sensitivity analyses results are contained in the Electronic Supplementary Data online.

## Discussion

We developed a semi-Markov decision analytic model to assess the cost-effectiveness and budget impact of fidaxomicin compared with vancomycin as the first-line treatment of patient subgroups with CDI at increased recurrence risk, from the German payer perspective. Based on a willingness-to-pay threshold of €50,000, fidaxomicin was found to be dominant (less costly and more effective) in patients with cancer and cost-effective in all of the other subgroups.

Despite the substantially higher acquisition cost of fidaxomicin compared with vancomycin, this is offset by the reduction in costs associated with treating recurrence and by the reduced hospitalization costs; therefore, fidaxomicin is cost-saving in the cancer subgroup and associated with incremental costs of €291 to €461 per patient in the other groups.

The deterministic sensitivity analyses showed that the key drivers of cost-effectiveness are recurrence, clinical cure rates, and CDI-attributable mortality. This type of sensitivity analysis has some limitations, including an evaluation of the impact of only a small number of parameters, and the lack of account for the potential interdependence between parameters. However, it is useful for assessing the key drivers of cost-effectiveness. The findings of the current deterministic analyses reflect the results of phase III trials, showing that fidaxomicin was associated with lower rates of recurrence and higher rates of sustained response/global cure rates than vancomycin in patients ≥65 years, those with a previous episode of CDI [3, 17], those with severe CDI at baseline [3, 17], those taking concomitant antibiotics [7], or with renal impairment [13] or cancer [12]. Differences were statistically significant (*p* ≤ 0.05) for both variables for those with cancer or taking concomitant antibiotics [12, 13], for recurrence in those ≥65 years or with severe CDI in one study [3] and with chronic kidney disease stage 2 in another [13] and for sustained response in those with severe CDI at baseline [17]. Moreover, analysis of the combined data for these trials showed that overall, fidaxomicin reduced persistent diarrhea, recurrence, or death by 40 % compared with vancomycin [30]. These effects of fidaxomicin are likely to reduce hospital readmission rates; indeed, in the current budget impact analysis, hospitalization costs were lower with fidaxomicin than vancomycin in all subgroups. A recent analysis of a patient discharge database showed that reductions in hospital-onset CDI and readmission of patients with an index CDI can provide tremendous cost savings to hospitals [31]. A reduced recurrence rate is likely in turn to reduce hospital readmission rates and the overall number of bed-days with fidoxamicin. Hospital bed-days have been reported to account for up to 94 % of the cost of CDAD treatment [32]. Furthermore, a recent real-world study showed that the overall readmission rate of fidaxomicin-treated patients was low (6.9 %) [33].

Cost-effectiveness in CDI treatment is important in the German setting as shown by a recent German hospital study that found CDAD generates a yearly overall cost of €464 million to the healthcare system [21]. In this study, recurring cases were associated with higher costs (€20,755 per case) than those with CDAD as a primary diagnosis (€4132) [21]. Recurring cases were associated with higher costs in a recent cost-of-illness analysis assessing the impact of CDAD and CDAD recurrence in the German health system [16]. In that analysis, the mean overall direct treatment costs in the recurrence group were €73,898 such that additional direct costs related to CDAD were €59,367 in the recurrence group compared with matched non-CDAD control patients [16].

Our analysis complements previous cost-effectiveness studies with fidaxomicin. A study from the perspective of Scottish public healthcare providers showed that fidaxomicin was cost-effective in patients with severe CDI and in those with a first CDI recurrence [18]. A US study from the third-party payer perspective found that fidaxomicin may be a more cost-effective option for the treatment of CDIs compared with vancomycin under most scenarios tested [34]. In addition, a recent cost-utility analysis from an Irish Health Service Executive perspective showed that fidaxomicin was dominant to vancomycin or metronidazole for the treatment of CDI [19]. Finally, a cost-utility study from the Spanish National Health Service perspective showed that the treatment of CDI with fidaxomicin would be cost-saving and lead to improved quality of life when compared with vancomycin in patients with cancer, renal impairment, or treated with concomitant antibiotics [35].

Limitations of the model include the need to make assumptions to address uncertainties. In the current model, the 6.5 % mortality rate was based on 30-day all-cause mortality from phase III trials. However, this is consistent with literature estimates for CDI-attributable mortality (5.7–6.9 %) [22–24]. Furthermore, in view of its more favorable sustained cure and recurrence rates, this is likely to be a conservative approach regarding the benefits of fidaxomicin vs. vancomycin. Indeed, it has been shown that recurrent CDI is associated with significantly higher mortality rates [36]. The model also applied the same recurrence risk to first and subsequent recurrence—again, this is a conservative assumption as in practice, the risk of a second recurrence is likely to be higher [37]. It was also assumed that all cases of CDI were treated on a general ward in hospital. In a real-world setting, it is likely that some patients with CDI will be treated in intensive care or an infectious diseases unit, while others may be treated in the outpatient setting. The assumptions for hospitalization costs and lengths of stay used in the model (€348/day on a general ward and 14.6 days) appear very conservative since a recent German hospital cost-of-illness study on the economic burden of CDAD [16] found that patients with recurrence spent 62 additional days in hospital (compared with those without recurrence), resulting in excess overall direct treatment costs of €55,438 per patient. Finally, another limitation of the model is that metronidazole was not included as a treatment option. This was because there are no direct comparative studies between fidaxomicin and metronidazole, although the results from a recent network meta-analysis indicate that fidaxomicin is associated with a significantly lower recurrence rate in CDI than metronidazole [38].

## Conclusion

Using a semi-Markov decision analytic model, our analysis in a German hospital setting showed that in patient subgroups with CDI at increased recurrence risk, first-line fidaxomicin was cost-effective vs. vancomycin, and less costly and more effective in patients with cancer over a 1-year time horizon. This is despite the higher acquisition cost of fidaxomicin and is a result of savings associated with lower hospital readmissions as more recurrences are prevented with fidaxomicin. These results are highly relevant given the emphasis on reducing hospital admissions and overall length of stay in European healthcare systems.

## Electronic supplementary material

Below is the link to the electronic supplementary material.

Supplementary material 1 (DOCX 37 kb)
