# Supplementary material for: A cost-effectiveness and budget impact analysis of first-line fidaxomicin for patients with Clostridium difficile infection (CDI) in Germany
Source: Infection. 2016 Apr 9;44(5):599–606. doi: 10.1007/s15010-016-0894-y (PMC5042976; doi:10.1007/s15010-016-0894-y)
Supplement: Supplementary file 1 — Supplementary material 1 (DOCX 37 kb) [file 15010_2016_894_MOESM1_ESM.docx]

**Electronic Supplementary data**

**Table 6** Deterministic sensitivity analysis - ≥1 recurrence

| Analysis | Change | Incremental cost | Incremental QALYs | ICER |
| --- | --- | --- | --- | --- |
| Base case |  | **€ 46,079** | **1.049** | **€ 43,929** |
| CDI-attributable mortality (fidaxomicin) | +20 % | € 43,467 | -0.089 | Fidaxomicin dominated |
|  | -20 % | € 48,693 | 2.192 | € 22,215 |
| CDI-attributable mortality (vancomycin) | +20 % | € 50,349 | 2.294 | € 21,950 |
|  | -10 % | € 41,786 | -0.206 | Fidaxomicin dominated |
| Probability of clinical cure (fidaxomicin) | +10 % | -€ 30,777 | 1.359 | Fidaxomicin dominates |
|  | -10 % | € 131,139 | 0.715 | € 183,415 |
| Probability of clinical cure (vancomycin) | +10 % | € 118,113 | 0.708 | € 166,735 |
|  | -10 % | -€ 32,640 | 1.391 | Fidaxomicin dominates |
| Probability of recurrence (fidaxomicin) | +20 % | € 94,223 | 0.744 | € 126,699 |
|  | -20 % | € 2,455 | 1.329 | € 1,847 |
| Probability of recurrence (vancomycin) | +20 % | -€ 43,366 | 1.723 | Fidaxomicin dominates |
|  | -20 % | € 120,436 | 0.474 | € 253,942 |
| Utility for patients with CDI (1st cycle) | +20 % | € 46,079 | 1.002 | € 45,971 |
|  | -20 % | € 46,079 | 1.096 | € 42,061 |
| Utility for CDI patients treated successfully (post-EOT) | +20 % | € 46,079 | 1.011 | € 45,576 |
|  | -20 % | € 46,079 | 1.087 | € 42,397 |
| Utility for CDI patients treated successfully (10-20 days post-EOT) | +20 % | € 46,079 | 1.028 | € 44,816 |
|  | -20 % | € 46,079 | 1.070 | € 43,077 |
| Utility for patients with no CDI | +20 % | € 46,079 | 1.364 | € 33,782 |
|  | -20 % | € 46,079 | 0.734 | € 62,788 |

CDI, *Clostridium difficile* infection; EOT, End of treatment; ICER, Incremental cost-effectiveness ratio; QALYs, Quality-adjusted life years

**Table 7** Deterministic sensitivity analysis – severe CDI

| Analysis | Change | Incremental cost | Incremental QALYs | ICER |
| --- | --- | --- | --- | --- |
| Base case |  | **€ 39,613** | **1.137** | **€ 34,845** |
| CDI-attributable mortality (fidaxomicin) | +20 % | € 37,579 | 0.028 | € 1,343,213 |
|  | -20 % | € 41,642 | 2.249 | € 18,512 |
| CDI-attributable mortality (vancomycin) | +20 % | € 43,550 | 2.365 | € 18,417 |
|  | -10 % | € 35,663 | -0.100 | Fidaxomicin dominated |
| Probability of clinical cure (fidaxomicin) | +10 % | -€ 32,561 | 1.428 | Fidaxomicin dominates |
|  | -10 % | € 118,714 | 0.821 | € 144,619 |
| Probability of clinical cure (vancomycin) | +10 % | € 108,889 | 0.823 | € 132,345 |
|  | -10 % | -€ 35,264 | 1.456 | Fidaxomicin dominates |
| Probability of recurrence (fidaxomicin) | +20 % | € 62,668 | 0.992 | € 63,159 |
|  | -20 % | € 17,650 | 1.275 | € 13,838 |
| Probability of recurrence (vancomycin) | +20 % | -€ 30,907 | 1.663 | Fidaxomicin dominates |
|  | -20 % | € 100,451 | 0.674 | € 149,080 |
| Utility for patients with CDI (1st cycle) | +20 % | € 39,613 | 1.089 | € 36,367 |
|  | -20 % | € 39,613 | 1.184 | € 33,445 |
| Utility for CDI patients treated successfully (post-EOT) | +20 % | € 39,613 | 1.092 | € 36,288 |
|  | -20 % | € 39,613 | 1.182 | € 33,512 |
| Utility for CDI patients treated successfully (10-20 days post-EOT) | +20 % | € 39,613 | 1.111 | € 35,657 |
|  | -20 % | € 39,613 | 1.163 | € 34,069 |
| Utility for patients with no CDI | +20 % | € 39,613 | 1.483 | € 26,713 |
|  | -20 % | € 39,613 | 0.791 | € 50,095 |

CDI, *Clostridium difficile* infection; EOT, End of treatment; ICER, Incremental cost-effectiveness ratio; QALYs, Quality-adjusted life years

**Table 8** Deterministic sensitivity analysis – concomitant antibiotics

| Analysis | Change | Incremental cost | Incremental QALYs | ICER |
| --- | --- | --- | --- | --- |
| Base case |  | **€ 29,080** | **0.948** | **€ 30,679** |
| CDI-attributable mortality (fidaxomicin) | +20 % | € 26,508 | -0.187 | Fidaxomicin dominated |
|  | -20 % | € 31,652 | 2.088 | € 15,159 |
| CDI-attributable mortality (vancomycin) | +20 % | € 33,079 | 2.177 | € 15,197 |
|  | -10 % | € 25,072 | -0.289 | Fidaxomicin dominated |
| Probability of clinical cure (fidaxomicin) | +10 % | -€ 46,311 | 1.249 | Fidaxomicin dominates |
|  | -10 % | € 111,593 | 0.624 | € 178,805 |
| Probability of clinical cure (vancomycin) | +10 % | € 96,106 | 0.655 | € 146,705 |
|  | -10 % | -€ 42,225 | 1.246 | Fidaxomicin dominates |
| Probability of recurrence (fidaxomicin) | +20 % | € 68,847 | 0.699 | € 98,509 |
|  | -20 % | -€ 7,611 | 1.180 | Fidaxomicin dominates |
| Probability of recurrence (vancomycin) | +20 % | -€ 31,216 | 1.388 | Fidaxomicin dominates |
|  | -20 % | € 82,391 | 0.552 | € 149,222 |
| Utility for patients with CDI (1st cycle) | +20 % | € 29,080 | 0.897 | € 32,419 |
|  | -20 % | € 29,080 | 0.999 | € 29,115 |
| Utility for CDI patients treated successfully (post-EOT) | +20 % | € 29,080 | 0.930 | € 31,279 |
|  | -20 % | € 29,080 | 0.966 | € 30,101 |
| Utility for CDI patients treated successfully (10-20 days post-EOT) | +20 % | € 29,080 | 0.939 | € 30,985 |
|  | -20 % | € 29,080 | 0.957 | € 30,378 |
| Utility for patients with no CDI | +20 % | € 29,080 | 1.216 | € 23,916 |
|  | -20 % | € 29,080 | 0.680 | € 42,775 |

CDI, *Clostridium difficile* infection; EOT, End of treatment; ICER, Incremental cost-effectiveness ratio; QALYs, Quality-adjusted life years

**Table 9** Deterministic sensitivity analysis – ≥65 years

| Analysis | Change | Incremental cost | Incremental QALYs | ICER |
| --- | --- | --- | --- | --- |
| Base case |  | **€ 46,116** | **1.036** | **€ 44,527** |
| CDI-attributable mortality (fidaxomicin) | +20 % | € 43,784 | -0.088 | Fidaxomicin dominated |
|  | -20 % | € 48,447 | 2.164 | € 22,390 |
| CDI-attributable mortality (vancomycin) | +20 % | € 50,095 | 2.266 | € 22,107 |
|  | -10 % | € 42,122 | -0.204 | Fidaxomicin dominated |
| Probability of clinical cure (fidaxomicin) | +10 % | -€ 28,380 | 1.335 | Fidaxomicin dominates |
|  | -10 % | € 128,086 | 0.712 | € 179,899 |
| Probability of clinical cure (vancomycin) | +10 % | € 116,032 | 0.715 | € 162,257 |
|  | -10 % | -€ 29,746 | 1.361 | Fidaxomicin dominates |
| Probability of recurrence (fidaxomicin) | +20 % | € 81,441 | 0.813 | € 100,177 |
|  | -20 % | € 13,288 | 1.245 | € 10,676 |
| Probability of recurrence (vancomycin) | +20 % | -€ 28,474 | 1.594 | Fidaxomicin dominates |
|  | -20 % | € 109,909 | 0.547 | € 200,747 |
| Utility for patients with CDI (1st cycle) | +20 % | € 46,116 | 0.990 | € 46,589 |
|  | -20 % | € 46,116 | 1.082 | € 42,640 |
| Utility for CDI patients treated successfully (post-EOT) | +20 % | € 46,116 | 0.998 | € 46,186 |
|  | -20 % | € 46,116 | 1.073 | € 42,983 |
| Utility for CDI patients treated successfully (10-20 days post-EOT) | +20 % | € 46,116 | 1.015 | € 45,440 |
|  | -20 % | € 46,116 | 1.056 | € 43,650 |
| Utility for patients with no CDI | +20 % | € 46,116 | 1.347 | € 34,245 |
|  | -20 % | € 46,116 | 0.725 | € 63,634 |

CDI, *Clostridium difficile* infection; EOT, End of treatment; ICER, Incremental cost-effectiveness ratio; QALYs, Quality-adjusted life years

**Table 10** Deterministic sensitivity analysis – cancer

| Analysis | Change | Incremental cost | Incremental QALYs | ICER |
| --- | --- | --- | --- | --- |
| Base case |  | **-€ 80,621** | **1.641** | **Fidaxomicin dominates** |
| CDI-attributable mortality (fidaxomicin) | +20 % | -€ 82,584 | 0.536 | Fidaxomicin dominates |
|  | -20 % | -€ 78,661 | 2.750 | Fidaxomicin dominates |
| CDI-attributable mortality (vancomycin) | +20 % | -€ 75,653 | 2.910 | Fidaxomicin dominates |
|  | -10 % | -€ 85,608 | 0.362 | Fidaxomicin dominates |
| Probability of clinical cure (fidaxomicin) | +10 % | -€ 153,483 | 1.936 | Fidaxomicin dominates |
|  | -10 % | € 130 | 1.319 | € 98 |
| Probability of clinical cure (vancomycin) | +10 % | -€ 12,765 | 1.356 | Fidaxomicin dominates |
|  | -10 % | -€ 151,502 | 1.925 | Fidaxomicin dominates |
| Probability of recurrence (fidaxomicin) | +20 % | -€ 52,809 | 1.464 | Fidaxomicin dominates |
|  | -20 % | -€ 106,814 | 1.809 | Fidaxomicin dominates |
| Probability of recurrence (vancomycin) | +20 % | -€ 159,184 | 2.201 | Fidaxomicin dominates |
|  | -20 % | -€ 12,866 | 1.148 | Fidaxomicin dominates |
| Utility for patients with CDI (1st cycle) | +20 % | -€ 80,621 | 1.557 | Fidaxomicin dominates |
|  | -20 % | -€ 80,621 | 1.725 | Fidaxomicin dominates |
| Utility for CDI patients treated successfully (post-EOT) | +20 % | -€ 80,621 | 1.603 | Fidaxomicin dominates |
|  | -20 % | -€ 80,621 | 1.679 | Fidaxomicin dominates |
| Utility for CDI patients treated successfully (10-20 days post-EOT) | +20 % | -€ 80,621 | 1.621 | Fidaxomicin dominates |
|  | -20 % | -€ 80,621 | 1.661 | Fidaxomicin dominates |
| Utility for patients with no CDI | +20 % | -€ 80,621 | 2.112 | Fidaxomicin dominates |
|  | -20 % | -€ 80,621 | 1.170 | Fidaxomicin dominates |

CDI, *Clostridium difficile* infection; EOT, End of treatment; ICER, Incremental cost-effectiveness ratio; QALYs, Quality-adjusted life years

**Table 11** Deterministic sensitivity analysis – renal impairment

| Analysis | Change | Incremental cost | Incremental QALYs | ICER |
| --- | --- | --- | --- | --- |
| Base case |  | **€ 33,403** | **1.240** | **€ 26,947** |
| CDI-attributable mortality (fidaxomicin) | +20 % | € 30,452 | 0.086 | € 354,217 |
|  | -20 % | € 36,349 | 2.398 | € 15,156 |
| CDI-attributable mortality (vancomycin) | +20 % | € 38,651 | 2.519 | € 15,344 |
|  | -10 % | € 28,130 | -0.051 | Fidaxomicin dominated |
| Probability of clinical cure (fidaxomicin) | +10 % | -€ 39,645 | 1.528 | Fidaxomicin dominates |
|  | -10 % | € 111,548 | 0.934 | € 119,441 |
| Probability of clinical cure (vancomycin) | +10 % | € 102,729 | 0.948 | € 108,401 |
|  | -10 % | -€ 38,925 | 1.527 | Fidaxomicin dominates |
| Probability of recurrence (fidaxomicin) | +20 % | € 66,360 | 1.040 | € 63,808 |
|  | -20 % | € 2,436 | 1.429 | € 1,705 |
| Probability of recurrence (vancomycin) | +20 % | -€ 55,285 | 1.873 | Fidaxomicin dominates |
|  | -20 % | € 108,624 | 0.690 | € 157,419 |
| Utility for patients with CDI (1st cycle) | +20 % | € 33,403 | 1.185 | € 28,187 |
|  | -20 % | € 33,403 | 1.294 | € 25,813 |
| Utility for CDI patients treated successfully (post-EOT) | +20 % | € 33,403 | 1.194 | € 27,983 |
|  | -20 % | € 33,403 | 1.285 | € 25,986 |
| Utility for CDI patients treated successfully (10-20 days post-EOT) | +20 % | € 33,403 | 1.214 | € 27,516 |
|  | -20 % | € 33,403 | 1.265 | € 26,402 |
| Utility for patients with no CDI | +20 % | € 33,403 | 1.613 | € 20,703 |
|  | -20 % | € 33,403 | 0.866 | € 38,586 |

CDI, *Clostridium difficile* infection; EOT, End of treatment; ICER, Incremental cost-effectiveness ratio; QALYs, Quality-adjusted life years

**Table 12** Proportion of patients who receive third line rescue treatment

| Subgroup | Proportion of fidaxomicin patients who have third line treatment | Proportion of vancomycin patients who have third line treatment |
| --- | --- | --- |
| All patients | 1.58 % | 2.40 % |
| At least 1 previous recurrence | 1.23 % | 1.71 % |
| Severe CDI | 4.21 % | 3.91 % |
| Concomitant antibiotics | 2.78 % | 7.39 % |
| Aged 65 years and above | 2.55 % | 3.18 % |
| Cancer | 2.41 % | 8.79 % |
| Renal failure | 7.39 % | 7.70 % |
